# Hyperdynamic circulation distinguishes predominantly diastolic hypertension from predominantly systolic hypertension

**DOI:** 10.1038/s41371-025-01069-7

**Published:** 2025-09-19

**Authors:** Tuomas P. Saarinen, Lauri J. Suojanen, Manoj Kumar Choudhary, Jukka Mustonen, Pasi I. Nevalainen, Jenni K. Koskela, Ilkka Pörsti

**Affiliations:** 1 Faculty of Medicine and Health Technology, Tampere University, Tampere, Finland; 2 Department of Internal Medicine, Tampere University Hospital, Tampere, Finland

**Keywords:** Hypertension, Blood flow

## Abstract

Elevated blood pressure is traditionally classified into systolic-diastolic hypertension, isolated systolic hypertension, and isolated diastolic hypertension. In this cross-sectional study, participants not using antihypertensive medications (n = 654) were divided into normotensive subjects (n = 421), and predominantly systolic (n = 130) versus predominantly diastolic hypertension (n = 103) based on the percentage elevation of aortic blood pressure above 125 mmHg systolic or 85 mmHg diastolic. Non-invasive hemodynamics were recorded using radial applanation tonometry and whole-body impedance cardiography during passive head-up tilt. Mean aortic blood pressures in the groups were 108/73, 141/89, and 131/94 mmHg, respectively. Mean age and BMI (43.6, 47.3 and 52.6 years; 25.9, 28.7 and 28.7 kg/m^2^, respectively) were lower in the normotensive than in hypertensive participants (p < 0.05). Predominantly systolic hypertension was characterized by higher forward wave amplitude, central pulse pressure, and systemic vascular resistance (p < 0.003 for all) than predominantly diastolic hypertension. Predominantly diastolic hypertension was characterized by higher heart rate and cardiac index (p < 0.004 for both), but lower stroke volume (p < 0.002), than predominantly systolic hypertension. Both hypertensive groups had increased systemic vascular resistance, but highest values were observed in predominantly systolic hypertension (p < 0.001). Pulse wave velocity was equally elevated by ~1 m/s in both hypertensive groups (p < 0.001). In response to head-up tilt, the increase in systemic vascular resistance, and the decrease in cardiac output, were more pronounced in predominantly systolic versus diastolic hypertension. To conclude, predominantly diastolic hypertension featured hyperdynamic circulation, while increased pulse pressure in predominantly systolic hypertension was related to higher stroke volume and systemic vascular resistance than in predominantly diastolic hypertension.

## Introduction

Hypertension is the most common cardiovascular disorder worldwide, while cardiovascular diseases are the leading cause of death globally [1, 2]. The pathophysiology of primary hypertension is multifactorial, involving genetic, environmental, and lifestyle factors. Together, these factors influence cardiovascular regulatory systems, leading to chronically elevated blood pressure (BP) and an increased risk of cardiovascular morbidity [1].

The primary hemodynamic determinants of BP are cardiac output and systemic vascular resistance [1]. Additionally, large arterial stiffness plays a key role in determining systolic BP and pulse pressure [3]. Traditionally, hypertension is classified into systolic-diastolic hypertension, isolated systolic hypertension, and isolated diastolic hypertension [4]. The hemodynamic profiles underlying these phenotypes are often simplified as follows: isolated systolic hypertension is associated with increased large arterial stiffness and elevated cardiac output, whereas isolated diastolic hypertension is linked to high systemic vascular resistance [5]. However, recent findings from a large cohort study of 33,414 participants suggest that these phenotypes exhibit heterogeneous hemodynamic profiles [6]. Overall, various combinations of cardiac output and systemic vascular resistance can contribute to elevated BP across all hypertensive categories [6].

We have previously used the orthostatic challenge to assess the functional hemodynamics of hypertension [7]. Postural changes alter autonomic nervous tone, systemic vascular resistance, and overall hemodynamics [7, 8]. Although BP is conventionally measured in the seated or supine position, daily human activities primarily occur in the upright position. Additionally, BP hyperreactivity to standing has been reported to be an independent predictor of adverse cardiovascular events [9].

Assessing the hemodynamic patterns underlying hypertension phenotypes may have future clinical implications, such as aiding in the personalization of treatment strategies [6]. This cross-sectional study aimed to investigate the hemodynamic patterns of predominantly systolic hypertension (PSH) and predominantly diastolic hypertension (PDH) by evaluating hemodynamic variables during passive head-up tilt (HUT).

## Subjects and methods

### Study population

The study included 654 normotensive and hypertensive individuals, all of whom participated in the DYNAMIC study (EudraCT 2006-002065-39, ClinicalTrials.gov registration NCT01742702). This investigation focuses on non-invasive recordings of hemodynamics in hypertensive patients compared with normotensive controls. The study was conducted in accordance with the Declaration of Helsinki, with approval obtained from the local Ethics Committee (code R06086M). All analyses and recordings followed the relevant guidelines and regulations.

Participants were recruited from four occupational health service units in the Pirkanmaa region. In addition, staff and students from Tampere University and Tampere University Hospital were invited through study announcements. Further recruitment was carried out via two local newspaper announcements and a newsletter distributed to individuals at the Varala Sports Academy. Written informed consent was obtained from all participants.

A medical doctor interviewed participants about their lifestyle, medications, underlying diseases, smoking habits, and alcohol consumption. Alcohol intake was assessed in terms of standard drinks (~12 g of absolute alcohol) per week. Office BP measurements were conducted according to the guidelines of the European Society of Hypertension [1]. Age, sex, smoking status, alcohol consumption, height, weight, and body mass index (BMI) were recorded. Participants taking medications that directly affect the cardiovascular system, including antihypertensives, were excluded. Exclusion criteria also included cardiovascular diseases other than hypertension, diabetes mellitus, chronic kidney disease, secondary hypertension, non-sinus rhythm, alcohol abuse (>24 restaurant doses per week), drug abuse, and psychiatric illnesses other than mild depression or anxiety.

A total of 156 control participants, 33 from the PDH group, and 49 from the PSH group reported regular medication use. The medications included: allopurinol (n = 3), androgen (1), antidepressants (41), antiepileptics (2), antihistamines (18), anxiolytics (5), acetylsalicylic acid (11), inhaled glucocorticoids (21), azathioprine (1), parenteral vitamin B_12_ (1), biological antirheumatics (1), inhaled beta2-mimetics (8), bisphosphonates (1), brinzolamide (1), calcium supplements (24), coxibs (3), vitamin D (103), digoxin (1), estrogen (17), topical estrogen (6), estrogen-progesterone combinations (50), ezetimibe (1), gabapentin (2), hypnotics (4), hormonal intrauterine devices (26), magnesium supplements (4), montelukast (1), non-steroidal anti-inflammatory drugs (NSAIDs) (5), oxychlorine (1), paracetamol (1), ocular prostacyclin analogues (3), potassium (4), proton pump inhibitors (16), prednisolone (1), progesterone (17), statins (17), retinoids (1), tamsulosin (3), thyroxine (22), sulfasalazine (3), tamoxifen (1), ocular timolol (2), triptans (1), varenicline (2), and warfarin (1). There were no significant differences in the use of other medications between study groups, except for a higher prevalence of NSAID use in the PSH group (n = 3) compared to controls (n = 1).

### Laboratory analyses

Laboratory analyses were performed at Fimlab Laboratories Oy Ltd, Tampere, Finland. Blood tests were taken after about 12 h of fasting, and a 12-lead electrocardiogram was recorded. Blood cell count was determined using ADVIA 120 or 2120 (Bayer Health Care, Tarrytown, NY, USA). Plasma concentrations of sodium, potassium, creatinine, cystatin C, C-reactive protein (CRP), uric acid, glucose, total cholesterol, high-density lipoprotein (HDL) and low-density lipoprotein (LDL) cholesterol and triglycerides were measured using Cobas Integra 700/800 (F. Hoffmann-Laroche Ltd., Basel, Switzerland) or Cobas6000, module c501 (Roche Diagnostics, Basel, Switzerland), and insulin concentrations using electrochemiluminescence immunoassay Cobas e411 (Roche Diagnostics, Basel, Switzerland). Nocturnal urine albumin excretion was determined using nephelometry (BN Prospec System, Siemens AG, Erlangen, Germany), and urine dipstick tests were analyzed using an automated refractometer (Siemens Clinitec Atlas or Advantus, Siemens Healthcare GmbH, Erlangen, Germany). Commercial kits were used to analyze plasma renin activity (GammaCoat^®^ Plasma Renin Activity 125˗I RIA Kit CA-1533, DiaSorin, Saluggia, Italy) and aldosterone concentration (Active^®^ Aldosterone RIA DSL-8600, Beckman Coulter, Fullerton, CA, USA). The aldosterone-to-renin ratio [10], quantitative insulin sensitivity check index (QUICKI) [11], and cystatin C based estimated glomerular filtration rate (eGFRcys) were calculated [12].

### Experimental protocol

Participants underwent a passive HUT protocol to record hemodynamics in both supine and upright positions [13]. These tests were conducted by research nurses in a temperature-controlled laboratory [14]. The total measurement duration was 10 min, consisting of 5 min in the supine position followed by 5 min of HUT at an angle of 60–65 degrees, during which data were continuously recorded. Average values for each minute were calculated. Participants were instructed to abstain from caffeine, smoking, and heavy meals for four hours, and from alcohol consumption for 24 h before the recordings.

### Non-invasive hemodynamics

Hemodynamics were recorded non-invasively using whole-body impedance cardiography and applanation tonometry [10, 13, 14]. A tonometric sensor (Colin BP-508, Colin Medical Instruments Corp., USA) was used to capture left radial BP and pulse waveform. The left upper arm was positioned on a support to maintain heart level in both the supine and upright positions. The signal was processed using SphygmoCor^®^ PWMx software (AtCor Medical, Australia) to calculate central and peripheral pulse pressure, pulse pressure amplification, aortic augmentation index (AIx), and aortic augmentation index standardized to 75 beats per minute (AIx@75) [10, 13, 14].

Whole-body impedance cardiography (CircMon^®^, JR Medical Ltd, Tallinn, Estonia) was used to determine heart rate, stroke volume, cardiac output, and aortic-to-popliteal pulse wave velocity (PWV) [15, 16]. Systemic vascular resistance was derived from cardiac output measured using CircMon^®^ and radial BP assessed with SphygmoCor^®^ [10]. Systemic vascular resistance, stroke volume, and cardiac output were adjusted for body surface area and presented as indexes: systemic vascular resistance (SVR) index, stroke index, and cardiac index, respectively [10]. We have found that stroke volume measured with CircMon^®^ correlates well with values obtained using three-dimensional ultrasound [15], and cardiac output values correlate well with those measured using thermodilution or the direct oxygen Fick method [16].

### Statistical analyses

**Fig. 1:**
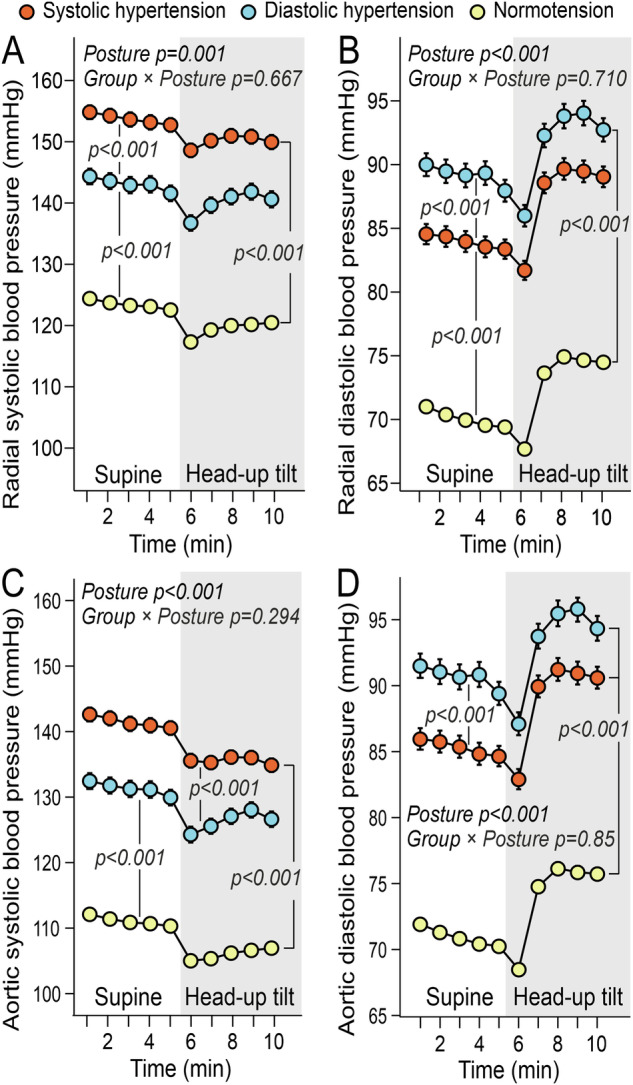
Blood pressure in laboratory measurements. Radial **A** and aortic **C** systolic blood pressure, and radial **B** and aortic **D** diastolic blood pressure during passive head-up tilt in subjects with predominantly systolic or diastolic hypertension and normotensive controls; adjusted for sex, age, BMI, and cystatin C; mean and standard error of the mean; statistics with generalized estimating equations.

Participants were classified into PDH (n = 103) and PSH (n = 130) groups based on the percentage elevation of aortic diastolic BP above 85 mmHg or systolic BP above 125 mmHg, respectively. These central BP thresholds approximate 135/85 mmHg at the brachial level [17, 18]. A percentage-based approach was used instead of absolute values, as systolic BP typically exhibits greater variability than diastolic BP (observed aortic ranges were systolic 80–177 mmHg; diastolic 50–114 mmHg). On average, aortic systolic BP was elevated by 4% (8%) in the PDH group and 13% (9%) in the PSH group, while aortic diastolic BP was elevated by 10% (8%) in the PDH group and 3% (9%) in the PSH group [mean (standard deviation, SD)] (Fig. 1). Participants with aortic systolic BP below 125 mmHg and diastolic BP below 85 mmHg served as normotensive controls (n = 421).

One-way analysis of variance (ANOVA) with a Bonferroni post-hoc test for normally distributed variables and the Kruskal-Wallis test with a post-hoc Dunn test for variables with skewed distribution were used to examine clinical characteristics and laboratory results. Functional circulatory data were analyzed using generalized estimating equations (GEE) to assess the influence of the study group, changes in the variable during HUT, and the interaction between group, posture, and hemodynamic variables — i.e., whether the change in the variable differed between groups in response to HUT. A linear scale response was applied with the autoregressive option for the correlation matrix, as successive measures of hemodynamics are highly correlated. The Bonferroni correction was applied in all GEE analyses.

The statistical analyses were adjusted for age, sex, BMI, and cystatin-C, which represent factors directly related to the level of BP [1]. Normally distributed variables are reported as mean and SD, or as mean and standard error of the mean (SEM), and non-normally distributed variables as median [25th–75th percentile]. Data were analyzed using SPSS 29.0 software (IBM SPSS, Armonk, NY, USA). The limit of statistical significance was set at *p*-value < 0.05.

## Results

### Demographics and laboratory analyses

The PDH group had the highest proportion of male participants (67%). Age differed between all groups: both hypertensive groups consisted of older participants than the normotensive group, with the oldest participants in the PSH group (Table 1). BMI and weight were higher in the hypertensive groups than in the normotensive group. There were no significant differences in the prevalence of smoking or alcohol consumption between the groups. Consistent with the group division based on tonometric recordings, BP values measured by physician and research nurse showed the highest diastolic values in the PDH group and the highest systolic values in the PSH group (Table 1).

**Table 1:** Demographics and clinical characteristics in subjects with predominantly systolic or diastolic hypertension and normotensive controls.

		Predominantly	
Variable	Normotension (*n* = 421)	Diastolic hypertension (*n* = 103)	Systolic hypertension (*n* = 130)
Males (%)	47.7	67.0*	48.5†
Age (years)	43.6 (11.8)	47.3 (9.9)*	52.6 (10.0)*†
Height (cm)	173.1 (9.7)	175.1 (8.6)	171.9 (8.4)†
Weight (kg)	78.2 (15.6)	88.0 (15.2)*	84.8 (14.7)*
Body mass index (kg/m^2^)	25.9 (4.1)	28.7 (4.4)*	28.7 (4.8)*
Current smokers (%)	13.5	13.6	9.2
Alcohol consumption (standard drinks/week)	2.0 [0.3–5.0]	3.0 [0.5–10.0]	3.0 [1.0–5.0]
Seated office blood pressure measured by physician			
Systolic (mmHg)	133 (17)	152 (17)*	160 (18)*†
Diastolic (mmHg)	85 (10)	100 (10)*	96 (9)*†
Supine office blood pressure measured by nurse			
Systolic (mmHg)	124 (12)	146 (14)*	152 (17)*†
Diastolic (mmHg)	76 (9)	93 (9)*	90 (8)*†

Results shown as mean (standard deviation) or median [25th, 75th percentile]; *p < 0.05 vs. normotension, †p < 0.05 vs. predominantly diastolic hypertension.

Blood hemoglobin and plasma sodium levels were higher in the PDH group compared to the normotensive group (Table 2). Plasma potassium, creatinine, and nocturnal urine albumin excretion were similar across all study groups. Total cholesterol, triglycerides, LDL-cholesterol, uric acid concentration, and cystatin C were higher, while eGFRcys was lower, in both hypertensive groups compared to the normotensive group. Uric acid concentration was highest in the PDH group. Both hypertensive groups had slightly higher fasting plasma glucose, with the PDH group also showing higher insulin levels compared to the normotensive group. The hypertensive groups also exhibited lower insulin sensitivity, as evaluated with QUICKI, compared to the normotensive group (Table 2). There were no differences in aldosterone levels between the groups. Plasma renin activity was lower, and the aldosterone-to-renin ratio was higher in the PSH group compared to both the PDH group and normotensive subjects (Table 2).

**Table 2:** Laboratory values in subjects with predominantly systolic or diastolic hypertension and normotensive controls.

		Predominantly	
Variable	Normotension (*n* = 421)	Diastolic hypertension (*n* = 103)	Systolic hypertension (*n* = 130)
Hemoglobin (g/l)	143 (12)	149 (11)*	145 (11)
Sodium (mmol/l)	140.2 (2.0)	141.1 (2.0)*	140.6 (2.1)
Potassium (mmol/l)	3.82 (0.28)	3.79 (0.33)	3.77 (0.28)
Creatinine (μmol/l)	74 (14)	77 (13)	73 (14)
Cystatin C (mg/l)	0.82 (0.15)	0.94 (0.12)*	0.90 (0.15)*
eGFRcys (ml/min/1.73 m^2^)	103 (18)	89 (15)*	90 (16)*
Nocturnal urine albumin (μg/min)^a^	4 [3–5]	4 [3–6]	4 [3–6]
C-reactive protein (mg/l)	0.7 [0.5–1.6]	1.4 [0.5–2.6]*	1.1 [0.5–2.4]*
Uric acid (μmol/l)	293 (73)	344 (83)*	317 (71)*†
Aldosterone (pmol/l)	443 [329–613]	437 [329–595]	434 [332–555]
Renin activity (ng Ang I/ml/h)	0.8 [0.5–1.4]	0.8 [0.5–1.4]	0.5 [0.3–1.0]*†
Aldosterone to renin ratio	579 [370–799]	593 [318–899]	770 [459–1217]*†
Total cholesterol (mmol/l)	4.97 (0.98)	5.47 (0.95)*	5.55 (1.09)*
Triglycerides (mmol/l)	0.98 [0.68–1.41]	1.27 [0.89–1.82]*	1.16 [0.93–1.66]*
HDL cholesterol (mmol/l)	1.61 (0.44)	1.42 (0.41)*	1.57 (0.46)†
LDL cholesterol (mmol/l)	2.86 (0.90)	3.48 (0.90)*	3.43 (0.95)*
Glucose (mmol/l)	5.4 (0.6)	5.7 (0.6)*	5.7 (0.6)*
Insulin (mU/l)	7.9 (6.0)	14.4 (39.5)*	9.4 (7.8)
QUICKI	0.362 (0.045)	0.341 (0.040)*	0.349 (0.033)*

Results shown as mean (standard deviation) or median [25th and 75th percentile]. eGFRcys, cystatin C based CKD-EPI formula, *HDL* high-density lipoprotein; *LDL* low-density lipoprotein, *QUICKI* quantitative insulin sensitivity check index;

*p < 0.05 vs. normotension;

**†***p* < 0.05 vs. predominantly diastolic hypertension;

^a^nocturnal albumin excretion was available from 359 (85%), 80 (78%), and 102 (78%) participants in the normotensive, predominantly diastolic, and predominantly systolic hypertensive groups, respectively.

### Hemodynamic results

The tonometric measurements, adjusted for sex, age, BMI, and cystatin C, showed the highest systolic BP in the radial artery and aorta in the PSH group (Fig. 1A, C) and the highest diastolic BPs in the PDH group (Fig. 1B, D). Radial and aortic BP responded similarly to HUT across all groups, with no significant group × posture interactions (Fig. 1).

**Fig. 2:**
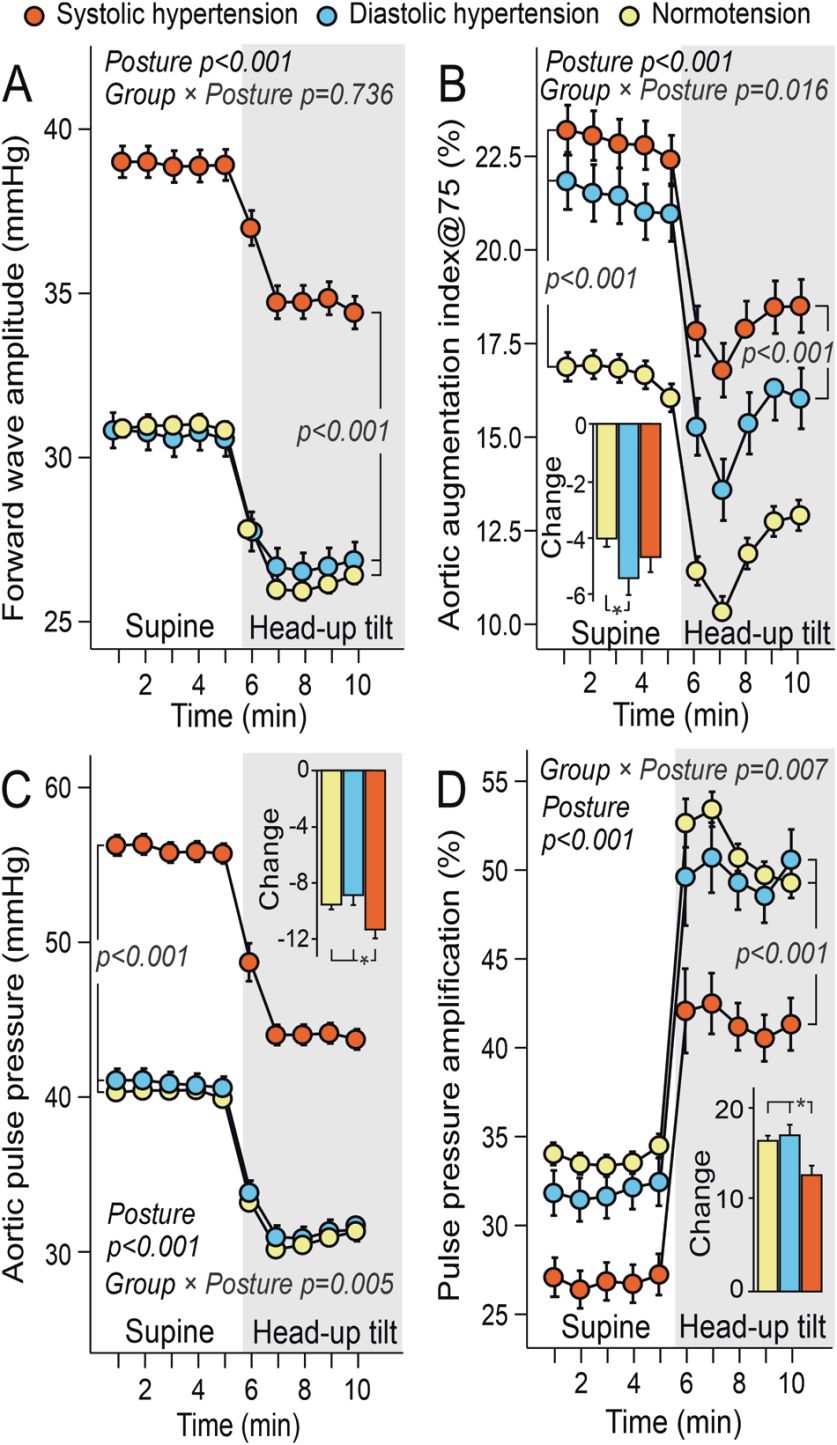
Pulse wave analysis results. Forward wave amplitude **A**, augmentation index related to heart rate 75/min **B**, aortic pulse pressure **C**, and pulse pressure amplification **D** during passive head-up tilt in subjects with predominantly systolic or diastolic hypertension and normotensive controls; adjusted for sex, age, BMI, and cystatin C; mean and standard error of the mean; statistics with generalized estimating equations.

Forward wave amplitude (Fig. 2A), AIx@75 (Fig. 2B), and aortic pulse pressure (Fig. 2C) were highest in the PSH group. AIx@75 was also elevated in the PDH group but was slightly lower than in the PSH group, while the HUT-induced decrease in AIx@75 was more pronounced in the PDH group than in the normotensive participants (Fig. 2B). Pulse pressure amplification was lowest in the PSH group (Fig. 2D). When shifting from a supine to an upright position, aortic pulse pressure decreased more (Fig. 2C) and pulse pressure amplification increased less (Fig. 2D) in the PSH group compared to the other groups. In the PDH group, pulse pressure >60 mmHg was observed in 21.3% (brachial office measurements), 12.6% (mean radial), and 0% (mean aortic) of participants. In contrast, pulse pressure >60 mmHg was observed in 48.5% (brachial office), 74.1% (mean radial), and 19.2% (mean aortic) of participants in the PSH group (p < 0.001 for all comparisons with the PDH group).

**Fig. 3:**
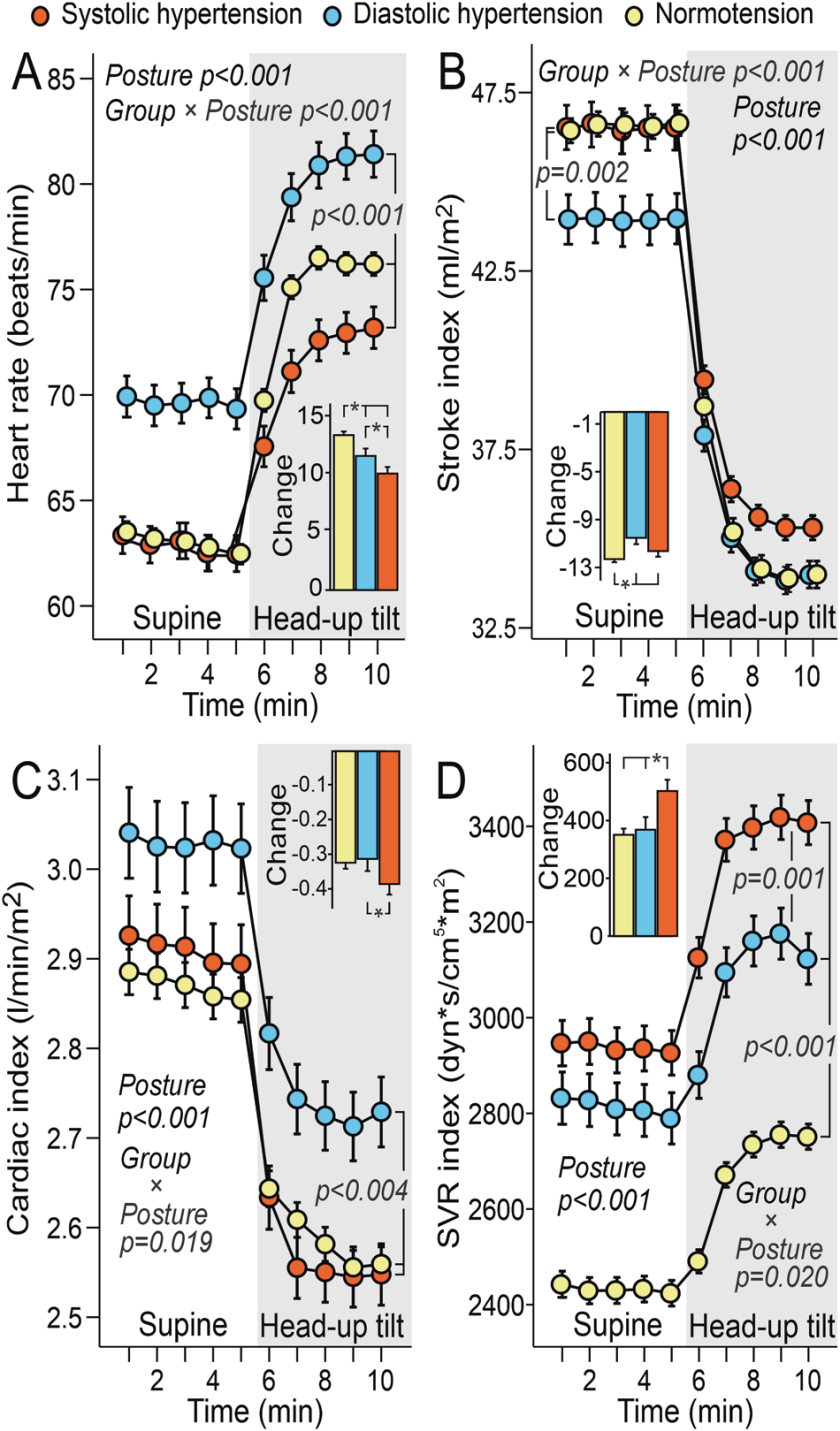
Cardiac variables and systemic vascular resistance. Heart rate **A**, stroke index **B**, cardiac index **C** and systemic vascular resistance (SVR) index **D** during passive head-up tilt in subjects with predominantly systolic or diastolic hypertension and normotensive controls; adjusted for sex, age, and cystatin C; heart rate also adjusted for BMI; mean and standard error of the mean; statistics with generalized estimating equations.

The PDH group had the highest heart rate (Fig. 3A) and cardiac index (Fig. 3C) in both supine and upright positions, while the normotensive group exhibited the greatest increase in heart rate during HUT. Stroke index was lowest in the PDH group in the supine position, whereas the normotensive group showed the greatest decrease in stroke index during HUT (Fig. 3B). SVR index was elevated in both hypertensive groups. In response to HUT, the PSH group exhibited the most pronounced decrease in cardiac index (Fig. 3C) and the greatest increase in SVR index (Fig. 3D).

**Fig. 4:**
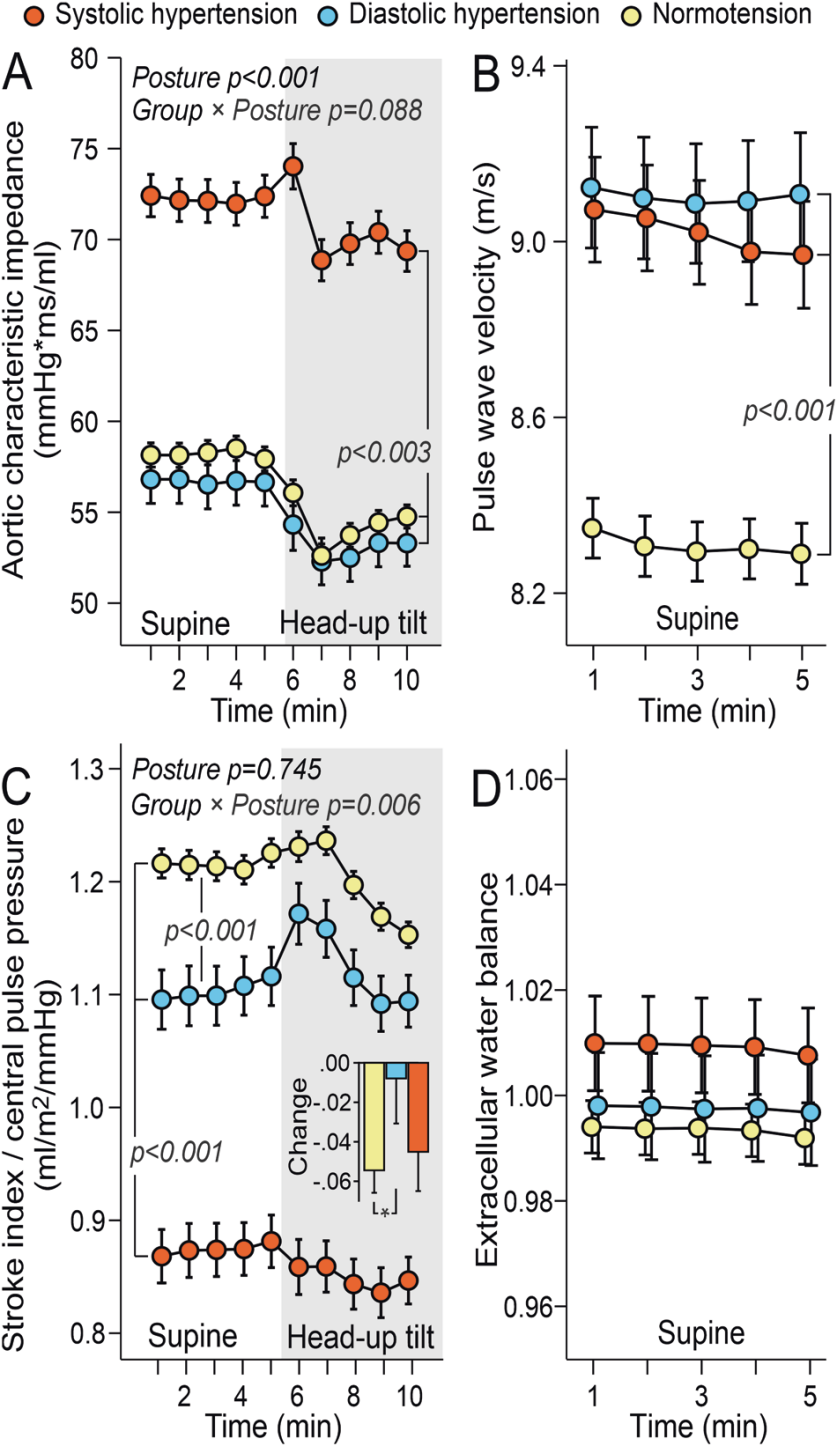
Aortic impedance, variables reflecting large arterial stiffness, and extracellular volume. Evaluated aortic characteristic impedance **A**, pulse wave velocity **B**, stroke index to central pulse pressure ratio **C** and extracellular water balance **D** in subjects with predominantly systolic or diastolic hypertension and normotensive controls; adjusted for sex, age, BMI, and cystatin **C**; mean and standard error of the mean; statistics with generalized estimating equations.

The evaluated aortic characteristic impedance was highest in the PSH group, with no differences between the PDH and normotensive groups (Fig. 4A). PWV was similarly elevated in both hypertensive groups (Fig. 4B). The ratio of stroke index to central pulse pressure, a variable reflecting large artery compliance [19, 20], was lowest in the PSH group and slightly lower in the PDH group than in the normotensive group (Fig. 4C). There were no differences in extracellular water balance among the groups (Fig. 4D).

If participants using any regular medications were excluded from the analyses (data not shown), all the above hemodynamic differences between the PDH and PSH groups remained statistically significant (with 68 and 85 individuals remaining in the groups, respectively).

## Discussion

In contrast to the traditional classification of hypertension into isolated systolic, isolated diastolic, and systolic-diastolic hypertension [4, 5], we investigated the hemodynamic differences between PDH and PSH by recording non-invasive hemodynamics in supine and upright positions. Participants were categorized into the PDH or PSH groups based on the percentage elevation of aortic BP above 85 mmHg diastolic or 125 mmHg systolic, respectively. Aortic BP was chosen for classification because central pressure is thought to be more strongly associated with vascular disease and mortality than brachial pressure [17, 18]. Additionally, a central BP of 125/85 mmHg closely corresponds to a brachial BP of 135/85 mmHg [17, 18]. In ambulatory BP recordings, 135/85 mmHg serves as the hypertension cut-off [1], while cut-off levels for radial tonometric BP in our laboratory are very similar [21]. The PDH group had the highest proportion of men, while both hypertensive groups were older, had higher BMI, and exhibited slightly lower eGFRcys than normotensive participants. Hemodynamic analyses were adjusted for these differences. Notably, BP measurements conducted by research nurses and physicians yielded consistent results, confirming the reliability of the tonometric BP measurements.

Both hypertensive groups exhibited less favorable metabolic profiles, characterized by higher total and LDL cholesterol, uric acid, and glucose concentrations in fasting plasma samples compared to normotensive controls. Insulin sensitivity, assessed using QUICKI, was also lower in both hypertensive groups—findings that are commonly observed in patients with hypertension [11, 22, 23]. In the PDH group, blood hemoglobin levels were higher, while HDL cholesterol concentrations were lower than in normotensive subjects. This group had the highest proportion of men, who typically have higher hemoglobin and lower HDL cholesterol levels than women [24]. Subsequently, these differences were no longer observed after adjusting for sex. Plasma aldosterone concentrations did not differ among the groups, but plasma renin activity was lowest in the PSH group, resulting in the highest aldosterone-to-renin ratio in this group.

Primary aldosteronism may affect over 10% of hypertensive patients [25]. Screening for primary aldosteronism is recommended if BP exceeds 150/100 mmHg in patients prone to hypokalemia [25]. In the present study, six patients from the PDH group and 23 patients from the PSH group met the screening criteria for primary aldosteronism. We previously found that bioimpedance recordings can detect extracellular fluid excess in patients with primary aldosteronism [10]. Similar extracellular water balance and plasma concentrations of potassium and aldosterone in all study groups suggest that the possible presence of primary aldosteronism had no major effects on the present results.

We examined hemodynamic regulation during passive HUT, as relatively few previous studies have addressed the cardiovascular responses to the postural change from supine to upright positions among hypertensive patients [9, 26, 27]. BP responses to upright posture vary, with some individuals showing an increase while others exhibit a decrease in BP [26]. Higher upright BP also influences patient prognosis. In the SPRINT study, approximately 21% of the participants exhibited a hypertensive orthostatic response (≥20 mmHg increase in systolic BP or ≥10 mmHg increase in diastolic BP), which increased the risk of cardiovascular events 1.44-fold when compared with participants without orthostatic hypertension [27]. Additionally, a 17-year follow-up study of more than 1200 young-to-middle-aged hypertensive subjects by Palatini et al. found that a greater increase in BP during postural change from supine to upright was associated with a higher risk of adverse cardiovascular events [9].

Increased SVR is considered a hemodynamic hallmark of essential hypertension [1]. However, elevated cardiac output is also a potential pathophysiological mechanism underlying BP elevation [7]. A large study utilizing impedance cardiography in over 34,000 participants with systolic BP ≥ 130 mmHg found that 70% exhibited high SVR, 15% showed high cardiac output, and 15% presented with a combination of these variables [6]. In the present study, SVR was elevated in both hypertensive groups when compared to normotensive subjects, regardless of whether they were supine or upright. However, the PSH group exhibited the greatest increase in SVR in the upright position. Wave reflection in the arterial tree is largely influenced by large arterial stiffness as well as the level of SVR [3, 28]. Since PWV did not differ between the two hypertensive groups, higher SVR likely explains the highest upright AIx@75 observed in the PSH group. Additionally, heart rate and cardiac output were highest in the PDH group, indicating the presence of hyperdynamic circulation. These findings align well with the recent hypothesis by Romero et al. suggesting that hyperdynamic circulation is a characteristic feature of isolated diastolic hypertension [5]. As the PDH group was characterized by higher SVR and higher cardiac output than those in the normotensive controls, the present results suggest that both of these hemodynamic characteristics should be taken into consideration when treating these patients. The present results do not allow conclusions about whether the alterations in SVR were functional or structural in nature.

Pulse pressure was evaluated at both aortic and radial levels. The peripheral-to-central pulse pressure ratio describes pulse pressure amplification, a beneficial cardiovascular variable that allows sufficient peripheral perfusion pressure to be achieved with lower central BP [3]. Elevated pulse pressure has been associated with higher mortality and an increased risk of cardiovascular events [29], while brachial pulse pressure exceeding 60 mmHg is included in the 2023 ESH criteria for hypertension-mediated organ damage [1]. Furthermore, reduced pulse pressure amplification is an independent risk factor for cardiovascular mortality and is also associated with large artery stiffness, a significant predictor of cardiovascular events [30, 31]. Typically, in isolated diastolic hypertension, pulse pressure is low due to the effect of higher diastolic pressure [5]. In the present study, the PSH group exhibited the highest pulse pressure that frequently exceeded 60 mmHg at the aortic and radial sites, along with the lowest pulse amplification and its increase during HUT. In contrast, the PDH group showed aortic pulse pressure and pulse pressure amplification comparable to those of the normotensive group. These findings regarding elevated pulse pressure in the PSH group align with existing evidence, supporting the increased risk of cardiovascular complications, particularly in systolic hypertension [1, 3, 17, 20].

PWV is the gold standard for assessing arterial stiffness [3, 17]. Large-artery stiffness is typically greater in isolated systolic than in isolated diastolic hypertension [3, 5, 17]. However, in the present study, PWV was similarly elevated in both hypertensive groups, indicating comparable stiffness in the PSH and PDH groups. In contrast, the stroke index–to–central pulse pressure ratio was lowest, and aortic characteristic impedance highest, in the PSH group. The latter reflects pulsatile afterload, is associated with aortic stiffness and smaller vessel diameter, and contributes to elevated aortic systolic pressure [32, 33]. Arterial compliance describes how much the artery stretches in total volume for a pressure increase, while arterial distensibility describes how much the artery stretches relative to its size for a pressure increase; with constant distensibility, a smaller vessel diameter yields lower compliance [34]. Thus, the higher forward wave amplitude, aortic pulse pressure, and aortic characteristic impedance in the PSH group, despite similar stroke volume and stiffness, suggest a smaller large-artery diameter compared with the PDH group.

The hemodynamic characteristics of the PDH and PSH groups are summarized in Table 3. Clinically, the PDH phenotype is associated with higher heart rates, whereas the PSH phenotype is linked to high pulse pressure and, if pulse wave analysis is available, lower pulse pressure amplification and higher forward wave amplitude.

**Table 3:** A summary comparing characteristics of predominantly diastolic hypertension (PDH) and predominantly systolic hypertension (PSH).

Similar in PDH and PSH	Pulse wave velocity
	Extracellular water balance
	Supine systemic vascular resistance
	Supine augmentation index
Higher in PDH	Heart rate
	Cardiac output
Higher in PSH	Forward wave amplitude
	Aortic pulse pressure
	Aortic characteristic impedance
	Upright augmentation index
	Upright systemic vascular resistance
Lower in PSH	Pulse pressure amplification

This study has some limitations. The classification of hypertensive patients into PDH and PSH groups led to some degree of phenotypic overlap. However, had the categorization been based on brachial BP, the overlap would have been greater due to variations in pulse pressure amplification [3]. The analyses were adjusted for demographic differences between the study groups, but residual confounding due to age, sex distribution, and BMI may still be present. Although participants were not taking medications with direct cardiovascular effects, the possibility of indirect influences from other medications cannot be ruled out. The present methodologies have been validated against invasive measurements and three-dimensional ultrasound [15, 16]. Nonetheless, the non-invasive assessments of stroke volume and cardiac output rely on mathematical modeling of the bioimpedance signal, which is a simplified version of the physiological processes [16]. Likewise, central BP was estimated using a mathematical approach applied to the radial artery tonometric signal [10, 13, 14]. The hemodynamic recordings were conducted over a 10-min period, offering a relatively short timeframe for assessment. However, compared to single-point BP and heart rate readings, this study analyzed over 600 cardiac cycles per participant. Thus, the minute-by-minute averages of all hemodynamic variables were derived from a considerable volume of data points. Although some concerns have been raised about the reliability of tonometric BP measurements [35], recent evidence from our research group indicates a strong correlation between these values and ambulatory daytime BP recordings in a cohort of 410 individuals [21]. Lastly, the cross-sectional design of this study precludes causal interpretations, highlighting the need for validation through longitudinal research.

In summary, the study provides insights into the hemodynamic profiles underlying PDH and PSH phenotypes. PDH featured hyperdynamic circulation, while PSH was associated with increased pulse pressure, higher stroke volume, aortic characteristic impedance, and SVR than PDH. These findings support the view that central arteries may have a smaller caliber in PSH than in PDH. Finally, the present results align with evidence supporting systematic measurement of BP and heart rate in supine and upright positions, in addition to the conventional seated BP measurements [7, 9, 26, 27].

## Summary

### What is known about the topic?

- Hypertension subtypes have distinct hemodynamic profiles.
- Isolated systolic hypertension is associated with increased large artery stiffness.
- Isolated diastolic hypertension is linked to elevated systemic vascular resistance.

### What this study adds?

- Predominantly diastolic hypertension features hyperdynamic circulation, whereas predominantly systolic hypertension shows elevated central pulse pressure and higher upright systemic vascular resistance.
- Reduced central artery caliber may contribute to predominantly systolic hypertension.
- Large artery stiffness is similar in predominantly systolic and diastolic hypertension.

## Data Availability

Analyses and generated datasets that support the current study are not available publicly. The datasets are available from the corresponding author on reasonable request.
